# MaAzaR, a Zn_2_Cys_6_/Fungus-Specific Transcriptional Factor, Is Involved in Stress Tolerance and Conidiation Pattern Shift in *Metarhizium acridum*

**DOI:** 10.3390/jof10070468

**Published:** 2024-07-04

**Authors:** Jinyuan Zhou, Siqin Wang, Yuxian Xia, Guoxiong Peng

**Affiliations:** 1 Genetic Engineering Research Center, School of Life Sciences, Chongqing University, Chongqing 401331, China; 2 Chongqing Engineering Research Center for Fungal Insecticide, Chongqing 401331, China; 3 Key Laboratory of Gene Function and Regulation Technologies Under Chongqing Municipal Education Commission, Chongqing 401331, China

**Keywords:** *Entomopathogenic fungi*, *MaAzaR*, microcycle conidiation, environmental tolerance

## Abstract

Entomopathogenic fungi are valuable sources of biological pesticides, with conidial yield and quality being pivotal factors determining their broad applications. AzaR, a fungus-specific zinc-cluster transcription factor, is known to regulate the biosynthesis of polyketone secondary metabolites in *Aspergillus niger*; however, its role in pathogenic fungi remains unclear. This study investigated the role of *MaAzaR* in the growth, development, and environmental tolerance of *Metarhizium acridum*. *MaAzaR* deletion slowed down conidial germination rate, caused reduction in conidial yield, lowered fungal tolerance to UV radiation, did not affect fungal heat-shock tolerance, and increased fungal sensitivity to the cell-wall-destructive agent calcofluor white. Furthermore, *MaAzaR* deletion transformed microcycle conidiation to normal conidiation on the microcycle conidiation medium. Transcription profile analysis demonstrated that *MaAzaR* could regulate transformation of the conidiation pattern by controlling the expression of genes related to cell division, mycelium growth and development, and cell wall integrity. Thus, this study identified a new gene related to fungal conidiation and environmental tolerance, enriching our understanding of the molecular mechanism of microcycle conidiation and providing theoretical support and genetic resources for the development of high-yielding strains.

## 1. Introduction

*Entomopathogenic fungi* serve as an optimal resource for pest management, characterized by their propensity to induce low levels of pest resistance and their environmental compatibility [1]. *Metarhizium acridum*, an entomopathogenic fungus, has been effectively utilized as a fungal insecticide to manage locust infestations in Africa, Asia, and Australia and serves as a prototypical organism for research on insecticidal strategies [2,3]. Conidia represent both the origin and terminal stages of differentiation within the fungal life cycle, having a critical function in the reproductive and survival mechanisms of fungi. Consequently, the capacity for conidiation and the quality of conidia are pivotal characteristics that govern the efficacy of large-scale production and deployment of mycopesticides [4]. In field applications, biopesticides still present issues such as their unstable protective effect due to environmental sensitivity and high costs, which constrain their widespread use. Therefore, investigation of the sporulation mechanism and stress resistance of *Entomopathogenic fungi* is crucial, representing a critical strategy for advancing the mycoinsecticides industry.

Fungal conidiation serves as a response to environmental modifications, and the vegetative growth of the fungi often stops and transits to conidiation under adverse conditions such as nutrient limitation [5], pH [6], or temperature stress [7]. In general, two modes of conidiation are observed in filamentous fungi, namely normal conidiation and microcycle conidiation [8]. Normal conidiation refers to the onset of conidiation following a protracted period of vegetative growth [9]. Microcycle conidiation functions as a survival strategy for certain fungi under detrimental conditions and is characterized by the absence or minimal growth of vegetative mycelium and immediate recurrence of conidiation following the germination of (sexual or asexual) conidia [9]. Anderson et al. observed that high-temperature exposure led to direct production of conidia in *Aspergillus niger* without associated hyphal growth, representing the initial documentation of microcycle conidiation [7]. Microcycle conidiation has been reported in over 100 fungal species, including *Penicillium*, *Alternaria*, etc. [5,7,9].

Extensive research has been conducted to elucidate the regulatory mechanisms of the normal conidiation pathway in *Aspergillus* spp., and the transcription factors (TFs) BrlA, AbaA, and WetA have been found to be involved in the fundamental regulatory pathway in *Aspergillus nidulans* [10,11]. In addition to the core regulatory pathway, various upstream activators and negative inhibitors, central regulatory factors, and light response and villus regulatory factors such as FluG, FlbA, FlbB, FlbC, FlbD, and FlbE [12,13,14,15] have been identified to function along with BrlA, AbaA, and WetA in controlling conidiation in *A. nidulans*. In contrast, studies on the molecular mechanism underlying microcycle conidiation are rather inadequate. When compared with normal conidiation, microcycle conidiation in *M. acridum* was noted to cause a faster conidial production rate, increased conidial yield, uniform conidial size, enhanced stress resistance, and superior conidial quality [16]. Several genes in *M. acridum* have been found to play a role in modulating conidiation patterns, including *MaH1* [17], *MaOpy2* [4], *MaNCP1* [18], etc.; however, the regulatory mechanisms controlling them remain elusive. Therefore, further investigation and identification of novel genes involved in microcycle conidiation and the molecular mechanisms governing microcycle conidiation are imperative, which can provide theoretical support and valuable resources for enhancing the fermentation processes and selecting high-yielding strains.

Numerous studies have demonstrated that various abiotic stress conditions, including UV radiation and high temperature, significantly constrain entomopathogenic fungi, limiting their field performance and virulence [19,20,21]. Temperature is a pivotal factor that affects the efficacy of *Entomopathogenic fungi* in controlling microbial pests, with heat stress particularly influencing the entire infection process [22]. The tolerance of fungi towards environmental stress is closely linked to the metabolic activities of specific biochemical components within their cells, such as protein [23] and trehalose [24]. Consequently, to sustain high efficiency of *Entomopathogenic fungi* in practical applications, it is crucial to enhance their UV and hygrothermal resistance. TFs are vital in signal transduction pathways and are key regulators of cellular functions [25]. Zinc-cluster TFs play pivotal roles as regulators in various biological processes in fungi and are involved in asexual sporulation, sexual morphogenesis, secondary metabolism, stress response, drug resistance, and pathogenesis [26,27,28,29]. For instance, BbTpc1 governs hyphal development and pathogenicity [30], whereas Pcz1 in *Penicillium roqueforti* is crucial for conidial formation and germination [31]. In *Aspergillus flavus*, 135 zinc-cluster TFs genes were identified to participate in critical processes such as hyphal growth and sporulation [32]. It was reported that AzaR (Azaphilone cluster-specific TF), a Zn2Cys6/fungus-specific TF, regulates the biosynthesis of polyketone secondary metabolites in *A. niger* [33]; however, our understanding of the functional role of AzaR is limited. Moreover, the *AzaR* gene homolog in the entomopathogenic fungus *M. acridum*, named *MaAzaR*, has not yet been characterized for its function. Therefore, in the present study, we examined the role of *MaAzaR* in conferring conidiation ability and stress resistance in *M. acridum*. The results revealed that *MaAzaR* is a novel gene related to conidiation and environmental tolerance of *M. acridum*, affecting the transformation of conidial formation patterns by regulating genes related to conidial formation and development.

## 2. Materials and Methods

### 2.1. Strains and Growth Conditions

*Metarhizium acridum* strain CQMa102, preserved in the China General Microbiological Culture Collection Center (Strain No. CGMCC 0877), was cultured on 1/4-strength Sabouraud’s dextrose agar (1/4 SDAY; 1% dextrose, 0.25% mycological peptone, 0.5% yeast extract, and 1.8% agar, *w*/*v*) at 28 °C.

### 2.2. Constructions of Mutants

The *MaAzaR* (Gene ID: MAC-06365) deletion mutant was constructed by homologous recombination. In brief, the upstream (about 1.2 kb) of the *MaAzaR* coding sequence was amplified by *MaAzaR*-LF and *MaAzaR*-LR, and the downstream flanking fragment (about 1.2 kb) was amplified by *MaAzaR*-RF and *MaAzaR*-RR and inserted into the pK2-PB vector harboring a bar cassette. The constructed pK2-PB-*MaAzaR*-LR vector was transformed into *M. acridum* using a *Agrobacterium tumefaciens*-mediated method. To rescue the deleted *MaAzaR*, the full-length *MaAzaR* sequence with flanking region (full length: 3.1 kb) was amplified and inserted into the pK2-*sur* vector containing the chlorimuron ethyl resistance gene *sur* [18] to construct the complementary vector pK2-*sur-MaAzaR*. The developed vector was transformed into Δ*MaAzaR*, and the complementary transformant (CP) was screened on Czapek medium (sucrose 30 g/L, NaNO_3_ 2 g/L, K_2_HPO_4_ 1 g/L, MgSO_4_·7H_2_O 0.5 g/L, KCl 0.5 g/L, FeSO_4_·7H_2_O 0.01 g/L, and agar 18 g/L) containing 60 μg/mL chlorsulfuron ethyl.

### 2.3. Growth Analysis

We prepared the conidia suspension of fungal strains according to the established method [18], using 0.05% Tween-80 aseptic solution and filtering through four layers of aseptic lens paper to remove potential mycelium. Then, the concentration of the conidia suspension was accurately determined by hematology analyzer. Next, the same amount of conidia suspension (1 × 10^7^ conidia/mL) was inoculated into 1/4 SDAY plates at 28 ℃. To monitor the germination process of the wild-type (WT), Δ*MaAzaR*, and CP strains, culture medium of equal amounts was taken out every 2 h and placed on a slide, and the conidia germination rate of different strains was counted under an optical microscope. In total, 100 conidia were counted each time, and the counting was repeated three times to calculate the germination rate of the conidia of different strains. This experiment was repeated three times. The conidial yield was assayed as previously described [18]. Specifically, the conidia suspensions of WT, Δ*MaAzaR*, and CP (1 × 10^6^ conidia/mL) were added to 24-well plates with 1 mL medium per well and cultured continuously at 28 °C for fifteen days. During this period, we washed the pore plate with 1 mL 0.1% Tween-80 solution every three days, collected conidia for counting, and accurately determined the spore production of each strain through the blood cell counter.

### 2.4. Stress Resistance Analysis

Fungal tolerance to UV irradiation and heat shock was assayed as previously described [4]. In brief, 50 µL of conidial suspension (1 × 10^7^ conidia/mL) of the WT, ∆*MaAzaR*, and CP strains were, respectively, inoculated onto 1/4 SDAY plates and subjected to either UV-B irradiation (1350 mW/m^2^) for 0, 1.5, 3.0, 4.5, and 6.0 h or incubated at 46 °C for 0, 3, 6, 9, and 12 h. Subsequently, the plates were incubated at 28 °C for 20 h, and their conidial germination rates were calculated using a hemocytometer as described earlier. Fungal tolerances to hyperosmotic and oxidative stresses were determined by spot assays on 1/4 SDAY plates with 1 M/L NaCl, 1 M/L sorbitol, and 6 mM/L H_2_O_2_, respectively. The fungal sensitivities to cell wall stressors were ascertained by spot assays on 1/4 SDAY plates with 0.01% *w*/*v* sodium dodecyl sulfate, 50 µg/mL calcofluor white (CFW), and 500 µg/mL Congo red. The conidial suspension (2 µL of 1 × 10^6^ conidia/mL) of the WT, ∆*MaAzaR*, and CP strains was, respectively, spotted on 1/4 SDAY without or with stressors and incubated for 6 days at 28 °C before photographing the fungal colonies.

### 2.5. Microscopic Observation of Conidiation

To observe the conidiation process of the fungal strains, the conidial suspension (100 µL of 1 × 10^7^ conidia/mL) was inoculated onto 1/4 SDAY plate and microcycle conidiation induction medium (SYA; 0.5% yeast extract, 3% sucrose, 0.05% MgSO_4_, 0.001% MnSO_4_, 0.05% KCl, 0.3% NaNO_3_, 0.1% KH_2_PO_4_, 0.001% FeSO_4_, and 2% agar, *w*/*v*) and incubated at 28 °C. After 10, 12, 18, 24, and 36 h of incubation, approximately 1 cm^2^ of the culture medium was cut for observation under a microscope (Motic, Guangzhou, China). The hyphal samples were stained with 10 µL of CFW (50 µg/mL) for 30 min after 18, 24, and 36 h of cultivation to visualize the mycelial septa under a fluorescent microscope (Nikon Eclipse Ci-E, Tokyo, Japan).

### 2.6. Determination of Trehalose Content

The trehalose content was extracted as previously described [34]. In brief, 0.1 g of mature conidia was cultured in 1/4 SDAY medium for 15 days. Subsequently, 1 mL of extraction liquid was added to the culture, and the conidia were crushed by ultrasonic wave, transferred to a centrifuge tube, allowed to stand at room temperature for 45 min, oscillated for 3–5 times, and centrifuged at 8000× *g* at room temperature for 10 min, and the supernatant was collected. The trehalose content in the supernatant was determined using a trehalose determination kit (Solarbio Biotechnology, Beijing, China) according to the manufacturer’s instructions.

### 2.7. RNA Sequencing

Transcriptional profiles of the WT and ∆*MaAzaR* strains were obtained using RNA sequencing (RNA-seq). In brief, the total RNA was extracted from the WT and ∆*MaAzaR* strains cultured on SYA plates at 28 °C for 10 h. The total RNA was extracted from each sample using an RNA kit (Invitrogen, Carlsbad, CA, USA) with RNase-free DNaseI and reverse-transcribed into cDNA with an oligo-dT primer using the PrimeScript RT Master Mix (TaKaRa, Dalian, China). Sequencing libraries were prepared, and sequencing was conducted on BGISEQ-500 (BGI, Beijing, China). The sequencing data were filtered using SOAPnuke for quality control to obtain clean reads and mapped to the reference genome of *M*. *acridum* using HISAT2 [35]. The differential splicing gene (DSG) was fused with Ericscript and rMATS. Clean reads were compared with the gene set by Bowtie2, and the gene expression level was calculated by RSEM. Basically, DESeq2 [36] was used for differential expression analysis, and the Q value was 0.05. In order to annotate DEGs, based on hypergeometric test, phyper was used for GO enrichment analysis (http://www.geneontology.org/ accessed on 14 February 2022). Bonferroni was used to correct the significance level (Q value of ≤0.05), which had a strict threshold. The DEGs were defined with a Q value of ≤0.005, and a fold change ≥1.5. RT-qPCR was used to verify the reliability of RNA-seq data. The RNA-seq data in this study were deposited in the NCBI BioProject database under the accession number No. PRJNA884297. Differentially expressed genes (DEGs) were classified and annotated using gene ontology (GO) and Kyoto Encyclopedia of Genes and Genomes (KEGG) pathway analysis.

### 2.8. Data Analysis

All experiments were performed in triplicate. Statistical analysis was performed using Statistical Package for Social Sciences (SPSS Inc., Chicago, IL, USA, version 26.0 for Windows). All the data are expressed as mean ± standard error. Student’s *t*-test was used for comparisons of normally distributed data between the groups, and values with *p* < 0.05 were considered as statistically significant.

## 3. Results

### 3.1. Characteristics of MaAzaR and Verification of Mutant Strain

**Figure 1:**
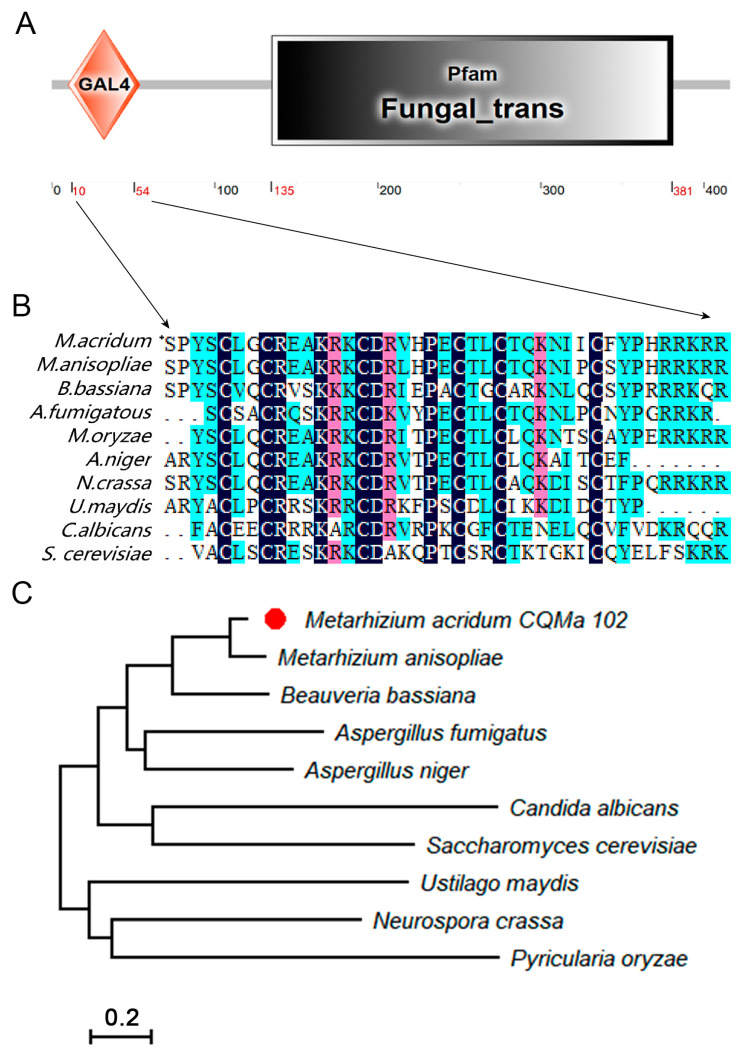
Structural and phylogenetic features of *MaAzaR*. (**A**) Domain structure analysis of *MaAzaR*. GAL4: GAL4-like Zn(II)2Cys6 binuclear cluster DNA binding domain; Fungal_trans: fungal TF regulatory middle homology region. (**B**) Domain sequence alignments of MaAzaR with other species. (**C**) Phylogenetic analysis of AzaR protein sequence from different fungi, including *Metarhizium anisopliae* (KAF5129833.1), *Metarhizium acridum* (CQMa102, XP_007812705.1), *Aspergillus fumigatus* (KAH1485999.1), *Beauveria bassiana* (XP_008596496.1), *Aspergillus niger* (GKZ68934.1), *Neurospora crassa* (XP_964064.3), *Ustilago maydis* (XP_011389956.1), *Saccharomyces cerevisiae* (NP_010031.1), *Canidia albicans* (KAF6072832.1), and *Pyricularia oryzae* (XP_003720741.1).

The *MaAzaR* coding sequence spans a total length of 1599 bp, including 348 bp introns encoding 416 amino acids with a molecular weight of 46.72 kDa. Prediction of structure domains using the online resource SMART (http://smart.embl.de/, accessed on 16 June 2021) revealed a Zn(II)2Cys6 bimodular cluster domain similar to GAL4 and a Fungal_trans domain (regulatory intermediate homology region of fungal TFs) (Figure 1A). Sequence comparison showed that the GAL4 sequence of Zn(II)2Cys6 zinc-cluster domain at MaAzaR was conserved, especially the Cys6 motif (Figure 1B). The sequence similarity was analyzed by DNAMAN version 8.0, and the phylogenetic tree was constructed by neighbor-joining method through MEGA-X version 10.1.8, which was tested by bootstrap (1000 repeats). The results indicated that MaAzaR exhibited high homology in filamentous fungi, with a closer relationship to *Metarhizium anisopiae* and significant sequence identity (Figure 1C).

**Figure 2:**
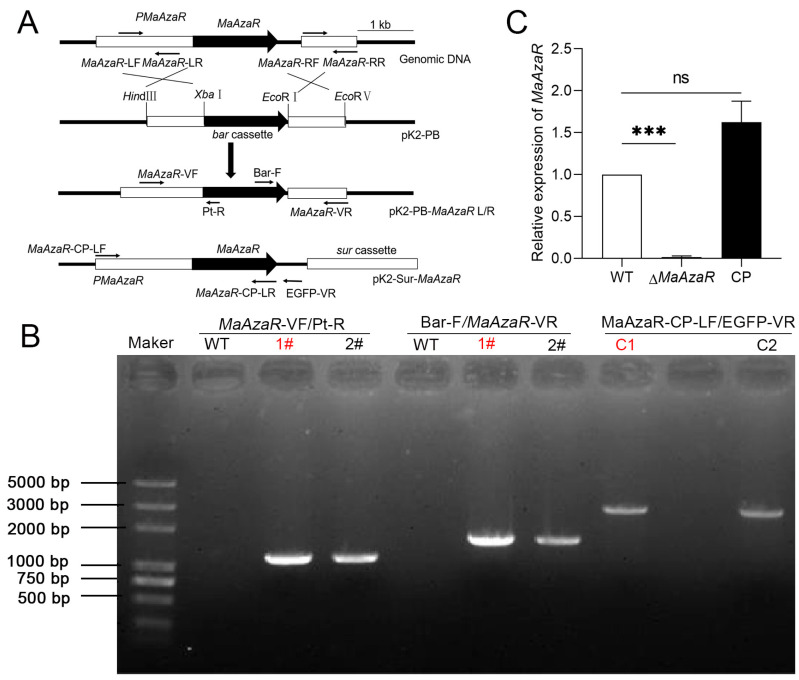
Construction of *MaAzaR* knockout and complementary vectors and verification of the corresponding strains. (**A**) Schematic diagram of the construction of the *MaAzaR* knockout and complementary vector; (**B**) PCR validation of knockout and complementary transformant. Δ*MaAzaR*-VF/Pt-R indicates left-arm validation of knockout transformant #1 and #2 (1323 bp); Bar-F/*MaAzaR*-VR denotes right-arm validation of knockout transformant #1 and #2 (1488 bp); *MaAzaR*-SF/GFP-VR indicates validation of complementary transformant C1 and C2 (3019 bp). The mutants used in subsequent experiments are marked in red font. (**C**) Analysis of *MaAzaR* gene expression in each strain. The expression level of *MaAzaR* in WT strain was used as a control. WT: wild type; Δ*MaAzaR*: *MaAzaR* knockout strain; CP: complementary strain. ***: *p* < 0.001; ns: no significant difference, *p* > 0.05. Error bars denote standard deviation for different strains.

Deletion and complementation vectors were developed through homologous recombination and random insertion strategy (Figure 2A). Successful knockout and recovery of the targeted genes were achieved through PCR amplification and quantitative verification (Figure 2B,C). In the subsequent experimental operation, we selected the knockout transformant 1# and the complementary transformant C1 for the experiment.

### 3.2. Disruption of MaAzaR Delayed Germination and Decreased Fungal Conidial Yield

**Figure 3:**
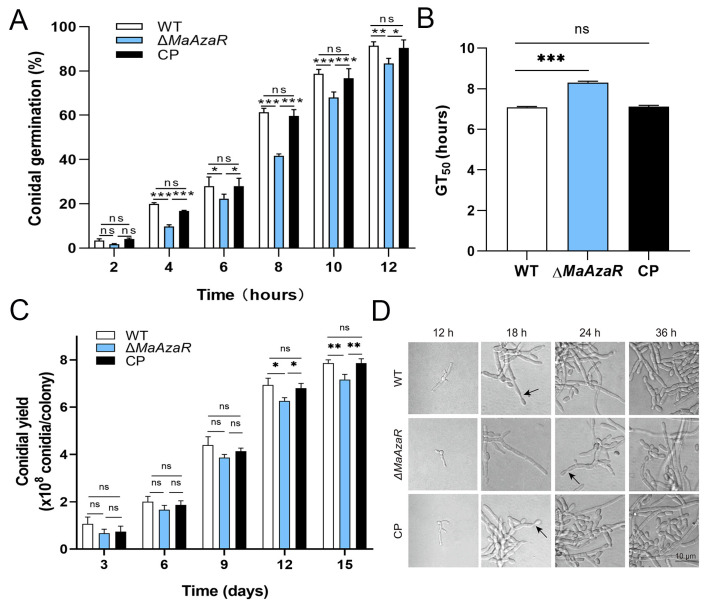
Characterization of growth and conidiation of the fungal strains on 1/4 SDAY medium. (**A**) Conidial germination rates (%) of the WT, Δ*MaAzaR*, and CP strains on 1/4 SDAY medium. (**B**) Analysis of GT_50_. (**C**) Conidial yields of the WT, Δ*MaAzaR*, and CP strains assessed on 1/4 SDAY medium. (**D**) Conidiation of the WT, Δ*MaAzaR*, and CP strains on 1/4 SDAY medium. Arrows indicate conidia on the conidiophores. Error bars denote the standard error of the mean. *: *p* < 0.05; **: *p* < 0.01; ***: *p* < 0.001; ns: no significant difference, *p* > 0.05.

To investigate the effect of *MaAzaR* on the growth and conidiation process of *M. acridum*, we first was evaluated the conidial germination rates of WT, Δ*MaAzaR*, and CP strains on nutrient-rich 1/4 SDAY solid medium (Figure 3A). When compared with the WT and CP strains, the germination rate of the Δ*MaAzaR* strain was extremely significantly lower at 4 h (*p* < 0.001). Further analysis of the median germination time (GT_50_) revealed that the GT_50_ of the Δ*MaAzaR* strain (8.02 ± 0.20 h) was extremely significantly higher than that of the WT (7.08 ± 0.36 h) and CP (7.11 ± 0.23 h) strains (*p* < 0.001; Figure 3B), indicating that the absence of *MaAzaR* delayed germination of *M. acridum*. Subsequently, the conidial yields were determined and found to be significantly reduced for the Δ*MaAzaR* strain when compared with those for the WT and CP strains (Figure 3C).

At 18 h, while the WT and CP strains were already showing obvious conidiation structures, the Δ*MaAzaR* strain was still in a mycelial growth state and did not produce conidiation structures until 24 h. At 36 h, while both the WT and CP strains presented significant conidial production, the Δ*MaAzaR* strain produced only very few conidia. These results consistently suggest that the absence of *MaAzaR* affected the growth and conidiation characteristics of CP, as evidenced by delayed germination, delayed formation of conidiation structures, and reduced conidial yields.

### 3.3. Disruption of MaAzaR Decreased Fungal Tolerance to UV-B Irradiation and Cell Wall Disrupting Agents

**Figure 4:**
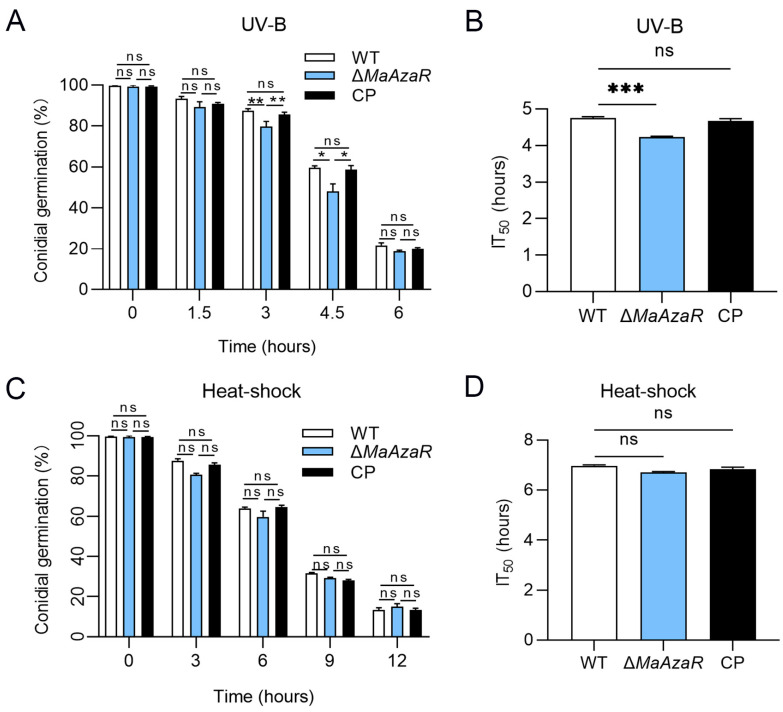
Assessment of UV-B irradiation and heat-shock tolerance. (**A**) Germination rates of the WT, Δ*MaAzaR*, and CP strains after 1.5, 3, 4.5, and 6 h of UV-B irradiation (1350 mW/m^2^). (**B**) Median inhibition time (IT_50_) of the WT, Δ*MaAzaR*, and CP strains after UV-B irradiation. (**C**) Germination rates of the WT, Δ*MaAzaR*, and CP strains after 46 °C heat-shock treatment for 3, 6, 9, and 12 h. (**D**) Median inhibition time (IT_50_) of the WT, Δ*MaAzaR*, and CP strains after damp heat treatment. Error bars indicate standard error of mean. *: *p* < 0.05; **: *p* < 0.01; ***: *p* < 0.001; ns: no significant difference, *p* > 0.05.

**Figure 5:**
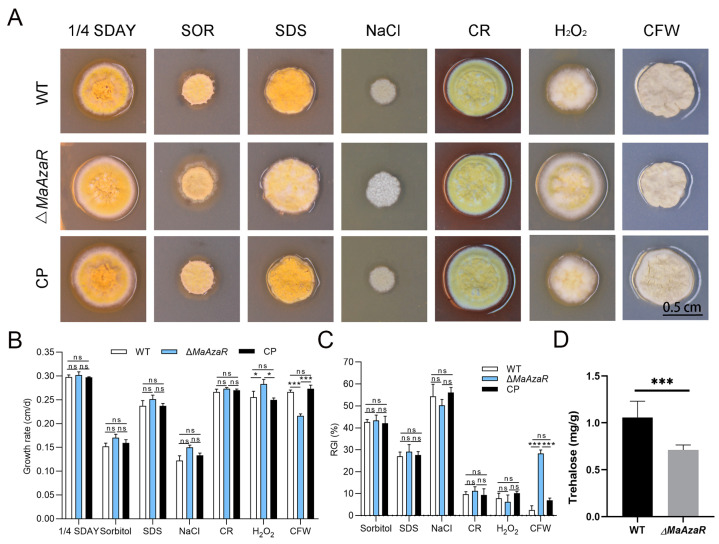
Chemical resistance analysis. (**A**) Colony growth status of each strain on various stress media supplemented with different chemical reagents. (**B**) Growth rate of each strain on different stress media. (**C**) Relative growth inhibition (RGI) of the fungal strains. (**D**) Intracellular trehalose accumulation in 15-day-old aerial conidia incubated on 1/4 SDAY. *: *p* < 0.05; ***: *p* < 0.001; ns: no significant difference, *p* > 0.05.

The conidial germination test was performed after UV-B radiation and heat-shock treatments. As shown in Figure 4A, the conidial germination rate of the Δ*MaAzaR* strain was significantly lower than those of the WT and CP strains after 3 h of UV-B radiation. Half-inhibition time analysis revealed that the Δ*MaAzaR* strain had a shorter half-inhibition time of about 0.5 h when compared with the WT and CP strains, indicating that the Δ*MaAzaR* strain was more sensitive to UV-B radiation than the WT and CP strains. In contrast, heat shock had no significant effect on the Δ*MaAzaR* strain (Figure 4C,D). Subsequently, we observed the growth of Δ*MaAzaR* strain in 1/4 SDAY medium supplemented with various types of chemical reagents in the presence of high salt content, oxygen, and osmosis as well as in the presence of fungal cell-wall- and cell-membrane-disrupting agents under adverse conditions. As shown in Figure 4A, colonies of the Δ*MaAzaR* strain grew slightly larger on the 1/4 SDAY medium without any added chemical reagents, with a slightly whitened periphery, loose folds in the center, and more developed mycelium, when compared with those of the WT and CP strains. In contrast, with the addition of the cell wall disruptor CFW, colonies of the Δ*MaAzaR* strain were considerably smaller and growth-restricted, presenting a significant slowdown in the growth rate (*p* < 0.001; Figure 5B) and significant increase in the relative growth inhibition rate (*p* < 0.001; Figure 5C) when compared with those of the WT and CP strains. This result suggests that the Δ*MaAzaR* strain is more sensitive to the cell wall disruptor CFW. Moreover, the intracellular trehalose content, which is important for fungal stress response, decreased to 32% in the Δ*MaAzaR* conidia when compared with that in the WT conidia (*p* < 0.01) (Figure 5D).

### 3.4. MaAzaR Regulates Conidiation Pattern Shift

**Figure 6:**
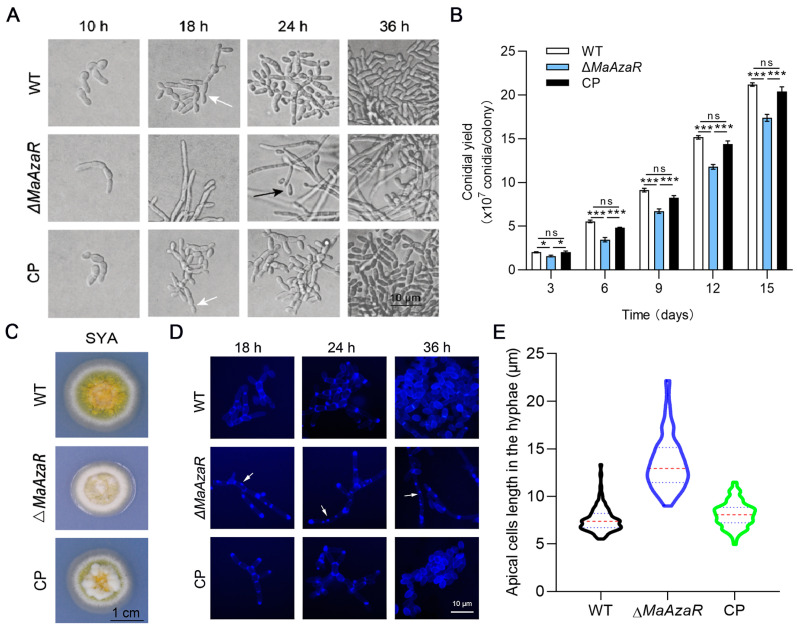
Characterization of growth and conidiation of different fungal strains on SYA medium. (**A**) Colony growth status of the WT, Δ*MaAzaR*, and CP strains after 5 days of growth on SYA medium (**B**) Conidial yields of Δ*MaAzaR*, WT, and CP strains on SYA medium. (**C**) Initial stage of conidiation of the WT, Δ*MaAzaR*, and CP strains on SYA medium (white arrow: microcycle conidiation; black arrow: normal conidiation). Error bars indicate standard error of mean. (**D**) Microscopic observation of chitin in hyphae stained with CFW. White arrows indicate irregular distributions of chitin. (**E**) Length of apical hyphal cells in the WT, Δ*MaAzaR,* and CP strains. *: *p* < 0.05; ***: *p* < 0.001; ns: no significant difference, *p* > 0.05.

To investigate whether *MaAzaR* affected the microcycle conidiation mode of *M. acridum*, the conidiation patterns of different fungal strains were observed on the SYA medium. As shown in Figure 6A, the Δ*MaAzaR* strain exhibited a shift in the conidiation mode. While microcycle conidiation commenced at 18 h in the WT and CP strains, the Δ*MaAzaR* strain grew as long hyphae without conidiation until 24 h. At 36 h, the conidia falling from the hyphal tips of the Δ*MaAzaR* strain were significantly fewer than those produced by the WT and CP strains through microcycle conidiation. Therefore, we speculated that deletion of *MaAzaR* might have reduced the conidial yield of *M. acridum*. From day 6 to day 15, the conidial yield of the Δ*MaAzaR* strain was significantly lower than those of the WT and CP strains (*p* < 0.001) (Figure 6B). Observation of the macroscopic colony status of each strain on the SYA medium revealed that Δ*MaAzaR* colonies were smaller and whitened, with a small amount of conidia on the surface, when compared with the WT colonies (Figure 6C). CFW staining revealed that *MaAzaR* contributed to chitin distribution in the hyphae. When compared with the WT and CP strains, chitin was not only distributed in the septa and tips of the hyphae but was also irregularly distributed in the extended hyphae of the Δ*MaAzaR* strain (Figure 6D). Moreover, disruption of *MaAzaR* also caused a significant increase in the apical cell length on the SYA medium (*p* < 0.001; Figure 6D,E).

### 3.5. Identification of DEGs Regulated by MaAzaR during Conidiation Pattern Shift Using RNA-seq

**Figure 7:**
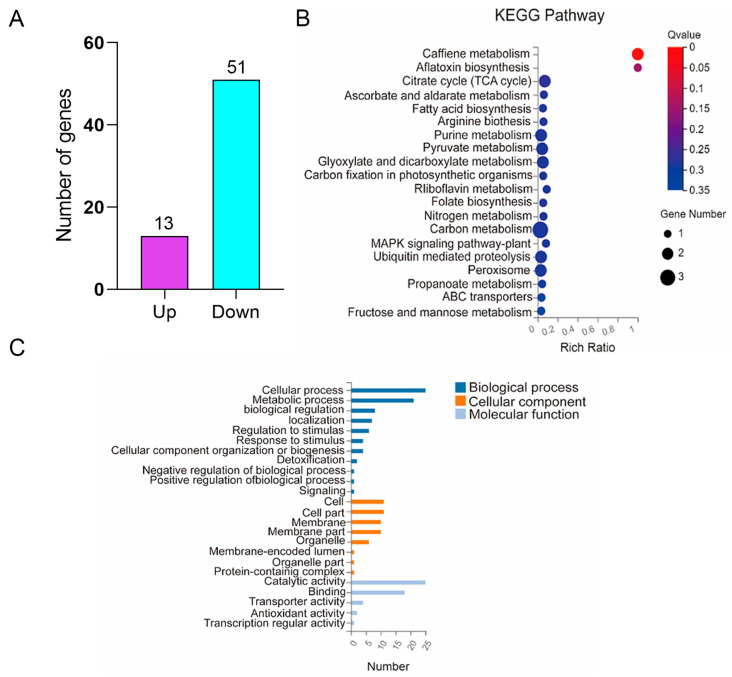
Number of DEGs in the conidiation stage transcriptome and analysis of the KEGG pathway and GO functional enrichment. (**A**) The number of upregulated and downregulated DEGs. (**B**) Enrichment analysis of DEGs in the KEGG pathway. (**C**) GO functional enrichment analysis of DEGs.

To elucidate the role of *MaAzaR* in conidiation pattern shift, the *MaAzaR*-regulated genes involved in conidiation pattern transition process were identified through RNA-seq (Accession No. PRJNA884297). Subsequently, WT and Δ*MaAzaR* samples cultured on SYA medium for 10 h were analyzed based on microscopic observations and expression patterns of *MaAzaR* during the conidiation pattern shift. A total of 64 DEGs were identified, among which 13 genes (20.3%) were upregulated, and 51 genes (79.7%) were downregulated (Figure 7A), which may include those involved in the conidiation pattern shift induced by *MaAzaR*. Furthermore, 15 DEGs (23.4%) were annotated as putative proteins (Table S1). To further validate the accuracy of DEGs, the upregulated and downregulated genes among the 64 DEGs were randomly selected for RT-qPCR verification. The results revealed that all the DEGs expressions were close to the RNA-seq data, and the expression trend was consistent, indicating that the RNA-seq data are valid (Figure S2). These findings further suggest the crucial role of *MaAzaR* in the conidiation pattern shift as well as the specific gene expression patterns associated with this process. KEGG pathway enrichment was performed on DEGs, and the enrichment bubble diagram shown in Figure 7B reveals that the DEGs were mainly enriched in various metabolic pathways, including carbon metabolism, purine metabolism, TCA cycle, and caffeine metabolism. The carbon metabolism pathway was the most enriched, and the caffeine metabolism pathway was the most significantly enriched.

Further GO functional enrichment analysis of the DEGs demonstrated that a total of 13 GO terms were enriched in DEGs, which could be classified into three major categories (Figure 7C). In the present study, the DEGs enriched for metabolic processes were predominantly concentrated in carbon metabolism, followed by cellular components involving the cell, cell membrane, etc., and molecular function including catalytic activity and binding. In summary, *MaAzaR* may influence the growth, development, and nutrient utilization of *M. acridum* by regulating the expression of genes related to cellular processes, components, metabolism, and catalytic functions, resulting in a switch in conidiation pattern.

## 4. Discussion

The results of the present study reveal a shift from microcycle conidiation to normal conidiation in the Δ*MaAzaR* strain in SYA medium, along with a significant reduction in conidial yield. To further explore the molecular mechanism of *MaAzaR* regulation of conidiation, we analyzed conidiation transcriptome data. The caffeine metabolism pathway was the most significantly enriched in KEGG pathway. Caffeine metabolism pathway-related proteins have been reported to be associated with DNA damage in yeast cells [37], cell wall integrity [38], and MAPK pathway [39]. GO functional enrichment analysis showed that the DEGs enriched for metabolic processes were predominantly concentrated in carbon metabolism, followed by cellular components involving the cell, cell membrane, etc., and molecular function including catalytic activity and binding. This result suggests that MaAzaR affects a variety of biological processes in the growth and conidiation of *M. acridum*. A previous study found that cellular processes can influence fungal cell differentiation and development as well as play an important role in cell growth and proliferation [40]. It was shown that genes related to membrane composition and structure can influence fungal spore morphology and mycelial development by affecting membrane formation and structure [41], while genes with catalytic activity were noted to influence nutrient uptake and utilization during fungal conidial growth [42]. Moreover, genes with cell-binding functions were found to regulate the binding and division of fungal conidia to influence conidiation [43].

Analysis of differential genes revealed that 64 genes were significantly differentially expressed (13 upregulated and 51 downregulated), 33 of which have been reported to be involved in key biological processes such as fungal growth and development, conidiation, stress response, cell division, and cell wall integrity. These DEGs included genes encoding enzymes such as catalase (MAC_03712), whose knockout in *C. albicans* was found to block hyphal development and reduce virulence [44], and phospholipid transport ATPase (MAC_07309), which was noted to be a core regulator of the growth, conidiation, and response to various stress stimuli of *B. bassiana* [45]. Furthermore, deletion of the adhesion protein gene MAD1 (MAC_00987) in *M. anisopliae* was observed to suppress conidia formation [46]. Additionally, the DEGs also included genes encoding TFs such as MADS-boxMEF2t-type TF (MAC_07653), which was reported to play an important role in nutrient utilization, sexual development, secondary metabolism, and virulence in *Fusarium verticillioides* [47], and the unique TF cutinase G box binding protein (MAC_01008) of Ascomycetes, whose deletion in *Ustilaginoidea virens* was found to affect fungal development and virulence [48]. Moreover, genes encoding the TF *MaSom1* (MAC_02477) downstream of the cAMP/PKA signaling pathway, which plays an important role in conidia formation and pathogenicity of *M. acridum* [49], were also found to be differentially expressed. These results suggest that *MaAzaR* regulates the expression of a variety of genes involved in fungal development during conidiation.

Among the 33 DEGs, 20 were downregulated, implying that *MaAzaR* mainly plays a negative regulatory role in the growth and conidiation processes in the Δ*MaAzaR* strain. A previous study indicated that deletion of *MaCreA*, a core regulator of carbon source metabolic blockade, in *M. acridum* significantly decreased conidiation by switching the conidiation mode from microcycle conidiation to normal conidiation [48]. Given that *MaAzaR* was downregulated and screened from a differential gene library for the switch in conidiation mode regulated by *MaCreA* in *M. acridum*, it can be presumed that *MaCreA* might regulate the expression of *MaAzaR*. Therefore, we searched for differential expression of genes associated with carbon source utilization in *MaAzaR* spore-producing transcriptome DEGs and identified that mannitol-1-phosphate 5-dehydrogenase (encoded by MAC_00279), which catalyzes the NAD(H)-dependent interconversion of D-fructose 6-phosphate and D-mannitol 1-phosphate in the mannitol metabolic pathway, and the key sugar isomerase-phosphoenolpyruvate carboxykinase (encoded by MAC_04725) were downregulated. Moreover, *MaAzaR*, in addition to regulating the expression of the abovementioned 33 DEGs, might also partly negatively regulate the expression of genes related to carbon source metabolism, inhibiting the nutrient utilization process of the strain and allowing the Δ*MaAzaR* strain to switch its conidiation pattern.

Stabilizing the effectiveness of the control is a major challenge for researchers studying entomopathogenic fungi. The survival of fungal conidia in the field is affected by exposure to abiotic factors such as high temperature, humidity, and UV-B radiation [50]. UV-B radiation was found to disrupt the cell wall and inactivate conidia in *B. bassiana* [51]. In the present study, a highly significant decrease in the conidial germination rate of the Δ*MaAzaR* strain was noted after 3 and 4.5 h of UV-B radiation, along with a significant reduction in the half-inhibition time, reduced tolerance to UV-B radiation, and unaffected hygrometric tolerance. The cell wall of filamentous fungi is a complex structure mainly composed of polysaccharides, primarily α-glucans, β-glucans, galactomannans, and chitins [46]. It is highly dynamic and plastic, possessing a heterogeneous composition that is pivotal for cell viability, morphogenesis, and pathogenesis. Furthermore, as the outermost layer, the cell wall serves as a protective barrier against environmental physical and chemical factors, regulating permeability and safeguarding cells from mechanical or osmotic stress [46]. In the present study, the Δ*MaAzaR* strain exhibited increased sensitivity to CFW. In particular, the growth of the Δ*MaAzaR* strain was constrained following supplementation of CFW to 1/4 SDAY medium, resulting in significantly smaller colonies when compared with those of the WT and CP strains. These observations are consistent with the findings reported by Bermejo et al., who demonstrated that CFW and other compounds interacting with cell wall components can lead to alterations in the cell wall, ultimately causing significant changes in cell morphology and appearance [52]. In *Saccharomyces cerevisiae*, application of CFW was found to cause the formation of abnormal septum, preventing the detachment and normal separation of daughter cells [53]. Studies in *B. bassiana* show that *BbpksP* impaired the cell wall structure of conidia and therefore UV tolerance [54]. Genes related to fungal cell wall composition, such as MAC_03926 (guanosine-diphosphatase) and MAC_03926 (guanosine-diphosphatase), were found in the RNA-seq database, which further explains the effect of the *MaAzaR* gene on the stress tolerance of *M. acridum* conidia. As a cell wall disruptor, CFW is known to exert its antifungal effect by binding to chitin components within the cell wall. In the present study, CFW staining of the hyphae evidently showed that the chitin distribution in the Δ*MaAzaR* strains was irregular. These abnormalities were not only confined to the mycelial tips and septum but were also observed as random distributions throughout the hyphae. This finding suggests that *MaAzaR* plays a pivotal role in regulating the growth and development of hyphae by influencing the distribution of chitin within the fungal cell wall.

## 5. Conclusions

In summary, the zinc-cluster TF MaAzaR, which is examined in the present study, plays a pivotal role in the biocontrol traits of *M. acridum*, including stress tolerance, conidiation ability, and conidiation mode. The results not only reveal the fundamental function of MaAzaR in *M. acridum* but also clarify the molecular mechanism underlying the conidiation mode shift. Thus, the present study provides a substantial theoretical foundation for the selection of *Entomopathogenic fungi* as well as the promotion and utilization of mycoinsecticides.

## Data Availability

RNA-seq data were deposited in the NCBI BioProject database (accession No. PRJNA884297).

## Supplementary Materials

The following supporting information can be downloaded at: https://www.mdpi.com/article/10.3390/jof10070468/s1, Figure S1. Transcription levels of *MaAzaR* on SYA medium. Figure S2. RT-qPCR validation of DEGs identified in RNA-seq analysis. Table S1. The DEGs in conidiation of Δ*MaAzaR* compared to WT. Table S2. Primers used in the study.
