# Supplementary material for: MaAzaR, a Zn2Cys6/Fungus-Specific Transcriptional Factor, Is Involved in Stress Tolerance and Conidiation Pattern Shift in Metarhizium acridum
Source: J Fungi (Basel). 2024 Jul 4;10(7):468. doi: 10.3390/jof10070468 (PMC11278141; doi:10.3390/jof10070468)
Supplement: Supplementary file 1 [file jof-10-00468-s001.zip › jof-3077607-supplementary.pdf]

MaAzaR, a Zn_2Cys_6/Fungus-Specific Transcriptional Factor, is Involved in Stress Tolerance and Conidiation Pattern Shift in *Metarhizium acridum*

--Supplementary Material

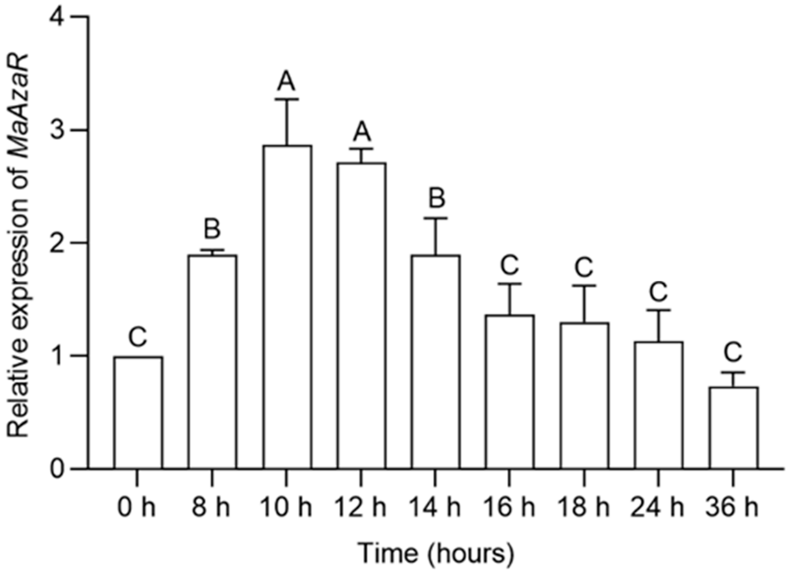

Figure S1 Transcription levels of *MaAzaR* on SYA medium.

A, B, C indicates $p < 0.001$, significant difference. *Error bars* indicate the standard deviation from the different strains.

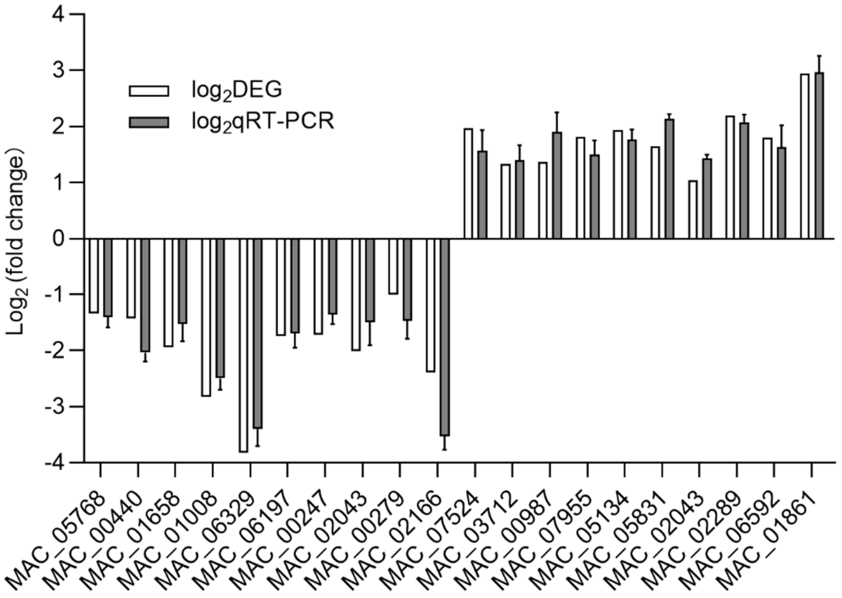

Figure S2. RT-qPCR validation of DEGs identified in RNA-seq analysis.

Table S1. The DEGs in conidiation of Δ*MaAzaR* compared to WT

Gene Symbol	log_2 (Δ*MaAzaR* / WT)	Qvalue (Δ*MaAzaR* / WT)	GeneBank Description
MAC_01861	2.943223856	0.0223783	Peptide synthetase
MAC_04761	2.706890342	0.007762494	Uncharacterized protein
MAC_00987	2.362449667	0.007762494	Adhesin protein MAD1
MAC_03712	2.328324588	2.56E-05	Catalase
MAC_02289	2.194740475	6.53E-04	Low affinity iron transporter, putative
MAC_07524	1.966844978	0.021938141	5 /3 -nucleotidase SurE family protein
MAC_05134	1.942785044	0.044479535	MFS domain-containing protein
MAC_07955	1.808905903	3.17E-04	Conidial pigment biosynthesis oxidase Mlac1
MAC_06592	1.802687824	0.0223783	Proline dehydrogenase
MAC_04211	1.73877539	0.025389643	6,7-dimethyl-8-ribityllumazine synthase
MAC_06371	1.703258295	0.026258965	Diphthine methyl ester synthase
MAC_05831	1.652095643	0.019088606	Chromosome segregation in meiosis protein
MAC_08357	1.643265845	0.009568745	Uncharacterized protein
MAC_02477	-1.543322696	0.037572897	WSC domain-containing protein
MAC_04600	-1.555658394	0.043797668	SOM1 protein
MAC_05269	-1.581428457	0.023163897	C2H2 type zinc finger domain-containing protein
MAC_05772	-1.581739519	0.02299074	Molybdenum cofactor sulfurase
MAC_04330	-1.610764774	0.035249111	[Histone H3]-trimethyl-L-lysine(9) demethylase
MAC_03173	-1.622570084	0.012799603	Calcium-related spray protein
MAC_04887	-1.633419766	0.009239832	PH domain protein
MAC_08058	-1.639355394	0.0223783	Putative aspartic endopeptidase
MAC_09532	-1.649324221	0.026082532	Oxoglutarate dehydrogenase (succinyl-transferring)
MAC_05649	-1.670843708	0.0223783	F-box and WD40 domain protein, putative
MAC_01054	-1.675633271	0.0223783	HECT-type E3 ubiquitin transferase
MAC_06341	-1.679191507	0.023981316	C2 domain containing protein
MAC_07347	-1.685520383	0.002812422	Uncharacterized protein
MAC_04397	-1.68812878	0.015197383	Vacuolar protein sorting-associated protein
MAC_07309	-1.692567182	0.009239832	Polynucleotide adenylyltransferase
MAC_03444	-1.712509037	0.0223783	Phospholipid-transporting ATPase

Gene Symbol	log2 (Δ*MaAzaR* / WT)	Qvalue (Δ*MaAzaR* / WT)	GeneBank Description
MAC_07653	-1.713120659	0.014787178	Cyclin N-terminal domain-containing protein
MAC_00918	-1.715177252	0.043797668	MADS-box MEF2 type transcription factor
MAC_01108	-1.729367838	6.53E-04	Prostaglandin G/H synthase 2/cyclooxygenase 2, pgh2/cox2, putative
MAC_03116	-1.733188858	0.009239832	Glutamine synthetase
MAC_06197	-1.742684944	0.007762494	Uncharacterized protein
MAC_00029	-1.778434095	3.17E-04	Putative SMP2 protein
MAC_03731	-1.798328347	0.001261456	Ribitol kinase
MAC_09259	-1.801361253	3.17E-04	Uncharacterized protein
MAC_04725	-1.82302064	1.18E-04	MDR efflux pump ABC3
MAC_06329	-1.8236482	0.018914093	Phosphoenolpyruvate carboxykinase (ATP)
MAC_01008	-1.833648734	0.005629726	Uncharacterized protein
MAC_05769	-1.852359861	0.007762494	Cutinase G-box binding protein
MAC_07175	-1.859366181	0.007762494	Conidial pigment biosynthesis oxidase Mlac1
MAC_00440	-1.868296735	0.012932274	Uncharacterized protein
MAC_03800	-1.878230324	0.001514532	Biotin carboxylase
MAC_00637	-1.896823978	1.72E-04	Sphingolipid long chain base-responsive protein
MAC_05425	-1.904730938	9.53E-05	Midasin
MAC_03001	-1.941721577	0.001486119	Uncharacterized protein
MAC_01658	-1.952064399	0.015197383	Ammonium transporter
MAC_03407	-1.958687965	0.001161671	Uricase
MAC_07182	-1.978272984	0.036474115	Uncharacterized protein
MAC_05768	-1.98117747	1.47E-05	Uncharacterized protein
MAC_04074	-1.994054305	7.27E-04	Xanthine dehydrogenase
MAC_01894	-2.004280802	9.17E-04	AAA family ATPase, putative
MAC_00279	-2.010395779	0.0223783	Flavin-containing amine oxidasedehydrogenase, putative
MAC_05927	-2.139642586	1.51E-04	Mannitol-1-phosphate 5-dehydrogenase
MAC_02571	-2.34314088	0.004583282	Uncharacterized protein
MAC_00250	-2.362769791	3.61E-12	Endoglucanase, putative
MAC_02166	-2.393386489	0.043797668	LEA domain containing protein

Gene Symbol	log2 (Δ*MaAzaR* / WT)	Qvalue (Δ*MaAzaR* / WT)	GeneBank Description
MAC_00249	-2.465990076	0.002869827	Uncharacterized protein
MAC_09120	-2.523665819	1.89E-06	LEA domain protein
MAC_09485	-2.683730113	0.029340517	Putative neurofibromin
MAC_00248	-2.706908518	0.0223783	Uncharacterized protein
MAC_02043	-2.719732752	3.17E-04	Uncharacterized protein
MAC_00247	-3.333700947	6.53E-04	Uncharacterized protein

Table S2 Primers used in the study

Primer	sequence(5′-3′)	Description
MaAzaR-LF	CTTCGCAATGCTGTCAAACT	construction and verification of *MaAzaR* vector
MaAzaR-LR	CGCCGTCTAAATACTACCCT	
MaAzaR-RF	GCCAGGACTCGGATTCATT	
MaAzaR-RR	AGAGCCCAGACCTCCACAT	
MaAzaR-VF	CCATGACGTTCTGCCAATAA	
MaAzaR-VR	GTTGAAGCTGCTGGTTCTGTG	
MaAzaR-SF	GGTTGTCAGCGTGAGGGA	
MaAzaR-SR	AAGCGGCTTGCAAAATCGACG	
Pt-R	CAGCCAAGCCCAAAAAGTG	
Bar-F	GCTCTACACCCACCTGCT	
GFP-VR	CGATGCGGTTCACCAGGGTGT	
MaAzaR-qF	CTACAAGACTCGTGGAACAA	Used for RT-qPCR analysis
MaAzaR-qR	GAAGCAGAGAAGGCATATTG	
Gapdh-qF	AGATGGAGGAGTTGGTGTTG	
Gapdh-qR	GACTGCCCGCATTGAGAAG	
